# Supplementary material for: Bcr4 Is a Chaperone for the Inner Rod Protein in the Bordetella Type III Secretion System
Source: Microbiol Spectr. 2022 Aug 30;10(5):e01443-22. doi: 10.1128/spectrum.01443-22 (PMC9603008; doi:10.1128/spectrum.01443-22)
Supplement: Supplemental file 1 — Text, Tables S1-S3, Fig. S1-S7. Download spectrum.01443-22-s0001.pdf, PDF file, 1.7 MB [file spectrum.01443-22-s0001.pdf]

Supplemental material

Text S. Supplementary Information

Fig. S1. Construction of the T3SS machinery in strains lacking Bcr4 and/or BspR. In the *B. bronchiseptica* wild-type (upper left), the BspR negative regulation level for the *bsc* locus transcription is moderate, and the T3SS machinery is established. In the Bcr4-deficient strain (upper right), BspR strongly represses the *bsc* locus transcription, and construction of the T3SS machinery is incomplete. In the BspR-deficient strain (lower left), the negative regulatory effect of BspR is cancelled, and the construction of the T3SS machinery is promoted. In the BspR/Bcr4 double-deficient strain (lower right), while the *bsc* locus transcription is promoted because of BspR deficiency, T3SS is not functional.

Fig. S2. Alignment of Bcr4 amino acid sequences in representative *Bordetella* species. Bcr4 amino acid sequences of *B. bronchiseptica* S798 (Bb), *B. pertussis* Tohama I (Bp) and *B. parapertussis* 12822 (Bp) were compared using ClustalW. The grey highlighted letters represent amino acid residues that were different from those in Bb. Bcr4 of Bp and Bpp have 98.3% and 97.1% identities with those of Bp, respectively.

Fig. S3. The nonspecific reaction of anti-BscI antibody to Bsp22. The culture supernatants (CS) were prepared from the wild-type strain, Δ*bsp22* (Bsp22-deficient strain),

Δ*bspR* (BspR-deficient strain) or Δ*bscI*+*bscI* (BscI-complemented strain) cultured in SS medium. The CS samples were separated by SDS-PAGE and stained with Coomassie Brilliant Blue (CBB, left panel) or analyzed by Western blotting (WB) with anti-BscI antibody (right panel). NS indicates nonspecific signals. Experiments were performed at least three times, and representative data are shown.

Fig. S4. The results of the RT-PCR analysis for the mRNA level of *bscI* in *B. bronchiseptica* strains. Total RNA was prepared from the wild-type strain, Δ*bspR* (BspR-deficient strain) or Δ*bspR*Δ*bcr4* (BspR- and Bcr4-deficient strain) cultured in SS medium and subjected to a quantitative RT-PCR analysis. The histogram shows the relative amount of *bscI* mRNA normalized by the housekeeping gene, *recA* mRNA. Experiments were performed at least three times, and representative data are shown.

Fig. S5. The results of the RT-PCR analysis for mRNA levels of *bopD* and *bscI* in the wild-type *B. bronchiseptica*. Total RNA was prepared from the wild-type strain cultured in SS medium and subjected to a quantitative RT-PCR analysis. The histogram shows the relative amount of *bopD* and *bscI* mRNA in the wild-type. The relative ratio of *bscI* mRNA is shown when the *bopD* mRNA amount is set as 1. Experiments were performed at least three times, and representative data are shown.

Fig. S6. The time course of BscI production in *B. bronchiseptica*. The whole cell lysates (WCL) were prepared from the wild-type strain, Δ*bcr4* (Bcr4-deficient strain), Δ*bspR* (BspR-deficient strain) or Δ*bspR*Δ*bcr4* (BspR- and Bcr4-deficient strain) cultured in SS medium for 0, 2, 5, 8 or 18 hr. The WCL were separated by SDS-PAGE and analyzed by Western blotting with antibodies against BscI, BopB and RpoB. Experiments were performed at least three times, and representative data are shown.

Fig. S7. The predicted structural model of *B. bronchiseptica* Bcr4

(A) The AlphaFold2 (AF2)-predicted structural model of *B. bronchiseptica* Bcr4 and structural comparison with *Aeromonas hydrophila* AcrH (PDB: 3WXX) and *Pseudomonas aeruginosa* PscG (PDB: 2UWJ). Z-score, root mean square deviation (RMSD), and amino acid identity (AA %ID) of AcrH and PscG compared with Bcr4 are shown, respectively. (B) The pairwise sequence alignment of Bcr4, AcrH, and PscG. The most frequent amino acid type is colored. The secondary structure assignments (H/h: helix, E/e: strand, L/l: coil) are also shown.

Table S1. Nomencalture of the *Bordetella* T3SS component

Table S2. Plasmids used in the study

Table S3. Primers used in the study

Supplementary Information

SI Materials and Methods

Pull-down assay.

The plasmids and primers used in this study are listed in Table S1 and S2, respectively. In order to express *bscI*, *bscK*, *bscJ* or *bcr4* tagged with a V5 sequence at the respective C-terminus (*bscI*-V5, *bscK*-V5, *bscJ*-V5 or *bcr4*-V5), we amplified DNA fragments encoding the *bscI*, *bscK*, *bscJ* or *bcr4* genes with the primer sets of B1-bscI-V5 and B2-bscI-V5, B1-bscK-V5 and B2-bscK-V5, B1-bscJ-V5 and B2-bscJ-V5 or B1-bcr4-comp and B2-bcr4-V5, respectively, using *B. bronchiseptica* S798 genomic DNA as the template. Each resulting PCR product was cloned into pDONR201 to obtain pMGKU404, pMGKU405, pMGKU406 or pMGKU407, respectively, by means of adapter PCR and site-specific recombination techniques using the Gateway cloning system (Invitrogen). Each plasmid was mixed with an expression vector such as p99*ccdB*-V5 (1) to obtain pMGKU408, pMGKU409, pMGKU410 or pMGKU411 using the Gateway cloning system.

BL21 cells carrying pMGKU408, pMGKU409 pMGKU410 or pMGKU411 were cultured overnight at 37ºC with shaking, and then diluted 1:40 in LB liquid medium containing 50 μL/mL ampicillin and incubated for 2 h at 37ºC with shaking. Each bacterial culture was further incubated for 5 h at 30ºC in the presence of isopropyl-beta-thiogalactopyranoside (IPTG) at the final concentration of 1 mM. Bacteria were collected by centrifugation at 2,600×*g* for 15 min, and suspended in cold TBS containing protease inhibitor cocktail,

cOmplete (Roche). Each bacterial suspension was sonicated, and each supernatant was used for the pull-down assay.

Next, in order to purify Bcr4 or BscI tagged with six histidine residues (6×His) at the respective N-terminus and Strep at the respective C-terminus, we amplified DNA fragments encoding *bcr4* or *bscI* with the primer sets of 5-HindIII-bcr4 and 3-bcr4-Strep, or 5-HindIII-bscI and 3-bscI-Strep using *B. bronchiseptica* S798 genomic DNA as the template. Each amplified DNA fragment was used as a template for 2nd PCR with a primer set consisting of the upper primer used in the 1st PCR and 3-Strep-HindIII to add a 24 bp sequence encoding the Strep tag. Each resulting PCR product was cloned into the HindIII recognition sites of pColdII to obtain pMGKU412 or pMGKU413 by the In-Fusion Cloning System (Clontech), respectively. In order to purify Bcr4 lacking the amino acids region 58–109 or 110–173, we amplified the DNA fragment with the primer sets of 5-bcr4-58-109-Strep-IF and 3-IF-bcr4-58-109-Strep, or 5-bcr4-110-173-Strep-IF and 3-IF-bcr4-110-173-Strep using pColdII-*bcr4*-Strep as the template. Each amplified fragment was self-ligated by the In-Fusion Cloning System, respectively, and then designated pMGKU414 or pMGKU415.

BL21 cells carrying pMGKU412, pMGKU413, pColdII-*bteA*-N-Strep (2), pMGKU414 or pMGKU415 were cultured overnight at 37ºC with shaking, and then diluted 1:100 in LB liquid medium containing 50 μL/mL ampicillin and incubated for 2 h at 37ºC with shaking, respectively. Each bacterial culture was further incubated overnight at 15ºC in the presence of IPTG at the final concentration of 0.05 mM. Bacteria were collected by centrifugation at

2,600×*g* for 20 min, and suspended in cold TBS containing cOmplete. Each bacterial suspension was sonicated and each supernatant except for that of pMGKU413 was subjected to purification using Ni-NTA agarose (Qiagen) according to the manufacturer's instructions. The purified proteins were dialyzed with TBS. As for pMGKU413, the bacterial suspension was sonicated and the pellet was suspended in Inclusion Body Solubilization Reagent (Thermo) according to the manufacturer's instructions. The BscI-Strep in solubilized solution was refolded as described previously (3). Briefly, the solubilized solution was diluted with 5-fold larger refolding buffer (20 mM Tris-HCl, pH-8, 150 mM NaCl, 10% glycerol) overnight at 4°C on a rotator, and then centrifugated at 20,000×*g* for 15 min. The supernatant was dialyzed with dialysis buffer (20 mM Tris-HCl, pH-8, 150 mM NaCl, 10% glycerol), and then centrifugated at 20,000×*g* for 15 min. The supernatant was used as purified protein for the pull-down assay.

We mixed the dual-tagged protein (6×His and Strep) and 30 *μ*l Strep-Tactin resin (IBA) in an Eppendorf tube and rotated the tube at 4°C for 1 h. Then, we washed the beads with TBS three times. Next, the V5-tagged protein-containing *E. coli* lysate was added to the tube and rotated at 4°C for 3 h. We transferred 30 *μ*L supernatant to new Eppendorf tube and added 30 *μ*L 2×SDS-PAGE sample buffer to prepare the Sup. Then we washed the beads with TBS (Fig. 1B and 1C) or TBS containing 0.1% Triton X-100 (Fig. 1C) three times and added 30 *μ*L 2×SDS-PAGE sample buffer to prepare the Pellet samples.

Construction of a *bscI* gene-disrupted or *bspR* /*bscI* double strains.

To construct the *bscI* or *bspR*/*bscI* double mutants, a 2.4 kb DNA fragment encoding *bscI* and its flanking regions was amplified by PCR with primers B1-bscI and B2-bscI using *B. bronchiceptica* S798 genomic DNA as the template. The resulting PCR product was cloned into pDONR201 to obtain pMGKU416 using the Gateway cloning system. An inverse PCR was carried out with the primers R1-bscI and R2-bscI using circular pMGKU416 as the template. The resulting PCR product was self-ligated using the In-Fusion Cloning System to obtain pMGKU417. This plasmid contained a 369-bp in-frame deletion from 30 bp downstream of the 5' end of the *bscI* gene to 30 bp upstream of the 3' end of the gene. This plasmid, pMGKU417, was mixed with a positive suicide vector such as pABB-CRS2 (4) to obtain pMGKU418 using the Gateway cloning system. The pMGKU418 or pABB-CRS2-*bspR* (5) plasmids in turn were introduced into *E. coli* Sm10λ*pir*, and transconjugated into the S798 wild-type or Δ*bscI* as described previously (6). The resulting mutant strains were designated Δ*bscI* or Δ*bspR*Δ*bscI*, respectively.

Construction of plasmids used for producing Bcr4 derivatives and BscI complementation

In order to produce full-length Bcr4 tagged with a FLAG sequence at the C-terminus, we performed an inverse PCR with primers of 5-bcr4-FLAG-IF and 3-IF-bcr4-FLAG using pDONR-*bcr4* (7) as the template. The resulting PCR product was self-ligated to obtain

pMGKU419 by the In-Fusion Cloning System. To produce Bcr4 lacking the amino acid regions 159–173, 164–173 or 169–173, inverse PCR was carried out with the primer sets of 5-*bcr4*-FLAG-IF and 3-IF-bcr4Δ169-173-FLAG, 5-*bcr4*-FLAG-IF and 3-IF-*bcr4*Δ164-173-FLAG, or 5-*bcr4*-FLAG-IF and 3-IF-*bcr4*Δ159-173-FLAG using pMGKU419 as the template. Each amplified fragment was self-ligated by the In-Fusion Cloning System to obtain pMGKU420, pMGKU421 or pMGKU422, respectively. For BscI complementation, a PCR was carried out with the primers of B1-bscI-V5 and 3-B2-bscI-comp using *B. bronchiceptica* S798 genomic DNA as the template. The resulting PCR product was cloned into pDONR201 to obtain pMGKU423 using the Gateway cloning system. These plasmids were mixed with pRK-R4-R3-F, pDONR-*fha*P and pDONR-*rrnB* (8) to obtain pMGKU424, pMGKU425, pMGKU426, pMGKU427 and pMGKU428 using the Gateway cloning system.

Quantitative reverse transcription-PCR

Total RNA was prepared from the *B. bronchiseptica* culture using a Trizol Max Bacterial RNA isolation Kit (Invitrogen), RNeasy Mini Kit (Qiagen), and RNase-free DNase Kit (Qiagen). The reverse transcription reaction was carried out using Transcriptor Universal cDNA Master (Roche) and T100 Thermal Cycler (Bio-Rad). The quantitative RT-PCR reaction was carried out using FastStart Essential DNA Probes Master (Roche) and Light Cycler 96 (Roche). To amplify the *bscI*, *bopD*, and *recA* genes, the primer sets 5-bscI and 3-bscI, 5-bopD and 3-bopD, 5-recA and 3-recA were used, respectively. For the experiment to determine the

presence of *bscI* mRNA in the Bcr4 mutant (Fig. S4), *recA* was used as an internal control. The values obtained from each strain were standardized with *recA*, and the relative amounts to the wild-type strain were determined. For the experiment to compare the amounts of *bscI* and *bopD* mRNA in the wild-type (Fig. S5), we followed the protocol provided by Roche. The genome of *B. bronchiseptica* was used as a template for quantitative RT-PCR to generate a calibration curve. The relative amount of *bopD* mRNA to *bscI* mRNA was then determined using the calibration curve.

Protein structure prediction

Protein structure prediction using amino acid sequences by AlphaFold2 with MMseqs2 (ColabFold) was performed on the Google Colab server with default parameters (9). A structure-based protein homology search using AlphaFold2-predicted structural models was performed on the Dali server (10). In addition, primary, secondary, and tertiary structures of query proteins were visualized and compared with those of neighbors on the Dali server.

References

1. Ogino T, Ohno R, Sekiya K, Kuwae A, Matsuzawa T, Nonaka T, Fukuda H, Imajoh-Ohmi S, Abe A. 2006. Assembly of the type III secretion apparatus of enteropathogenic *Escherichia coli*. J Bacteriol 188:2801-11.
2. Kuwae A, Momose F, Nagamatsu K, Suyama Y, Abe A. 2016. BteA Secreted from the *Bordetella bronchiseptica* Type III Secetion System Induces Necrosis through an Actin Cytoskeleton Signaling Pathway and Inhibits Phagocytosis by Macrophages. PLoS One 11:e0148387.
3. Halder PK, Roy C, Datta S. 2019. Structural and functional characterization of type three secretion system ATPase PscN and its regulator PscL from *Pseudomonas aeruginosa*. Proteins 87:276-288.
4. Sekiya K, Ohishi M, Ogino T, Tamano K, Sasakawa C, Abe A. 2001. Supermolecular structure of the enteropathogenic *Escherichia coli* type III secretion system and its direct interaction with the EspA-sheath-like structure. Proc Natl Acad Sci U S A 98:11638-43.
5. Kurushima J, Kuwae A, Abe A. 2012. The type III secreted protein BspR regulates the virulence genes in *Bordetella bronchiseptica*. PLoS One 7:e38925.
6. Donnenberg MS, Kaper JB. 1991. Construction of an eae deletion mutant of enteropathogenic *Escherichia coli* by using a positive-selection suicide vector. Infect Immun 59:4310-7.

7. Nishimura R, Abe A, Sakuma Y, Kuwae A. 2018. *Bordetella bronchiseptica* Bcr4 antagonizes the negative regulatory function of BspR via its role in type III secretion. Microbiol Immunol 62:743-754.

8. Kuwae A, Matsuzawa T, Ishikawa N, Abe H, Nonaka T, Fukuda H, Imajoh-Ohmi S, Abe A. 2006. BopC is a novel type III effector secreted by *Bordetella bronchiseptica* and has a critical role in type III-dependent necrotic cell death. J Biol Chem 281:6589-600.

9. Mirdita M, Schutze K, Moriwaki Y, Heo L, Ovchinnikov S, Steinegger M. 2022. ColabFold: making protein folding accessible to all. Nat Methods 19:679-682.

10. Holm L. 2020. Using Dali for Protein Structure Comparison. Methods Mol Biol 2112:29-42.

Table S1. Nomencalture of the *Bordetella* T3SS				
Unified name	*Bordetella*	*Yersinia*	*Pseudomonas*	Predicted function
SctF	BscF	YscF	PscF	Needle
SctI	BscI	YscI	PscI	Inner rod
SctE	BopB	YopB	PopB	Translocation pore
SctB	BopD	YopD	PopD	Translocation pore
SctA	Bsp22	LcrV	PcrV	Needle tip or Filament
SctJ	BscJ	YscJ	PscJ	Inner membrane ring
SctK	BscK	YscK	PscK	ATPase cofactor
SctN	BscN	YscN	PscN	ATPase

Table S2. Plasmids used in the study

Name	Description	Reference or source
pDONR201	DNA cloning vector, Km^r	Invitrogen
pMGKU404	pDONR201, *bscI* gene tagged with V5 sequence at the C terminus	This study
pMGKU405	pDONR201, *bscK* gene tagged with V5 sequence at the C terminus	This study
pMGKU406	pDONR201, *bscJ* gene tagged with V5 sequence at the C terminus	This study
pMGKU407	pDONR201, *bcr4* gene tagged with V5 sequence at the C terminus	This study
p99*ccdB*-V5	Expression vector for V5 tagged gene, Amp^r	1
pMGKU408	p99-*ccdB*-V5, *bscI* gene tagged with V5 sequence at the C terminus	This study
pMGKU409	p99-*ccdB*-V5, *bscK* gene tagged with V5 sequence at the C terminus	This study
pMGKU410	p99-*ccdB*-V5, *bscJ* gene tagged with V5 sequence at the C terminus	This study
pMGKU411	p99-*ccdB*-V5, *bsc4* gene tagged with V5 sequence at the C terminus	This study
pColdII	Expression vector for His tagged gene, Amp^r	TAKARA
pMGKU412	pColdII, *bcr4* gene tagged with Strep sequence at the C terminus	This study
pMGKU413	pColdII, *bscI* gene tagged with Strep sequence at the C terminus	This study
pMGKU414	pColdII, *bcr4* gene lacking amino acids region 58-109 tagged with Strep sequence at the C terminus	This study
pMGKU415	pColdII, *bcr4* gene lacking amino acids region 110-173 tagged with Strep sequence at the C terminus	This study
pColdII-*bteA*-N-Strep	pColdII, *bteA* gene coding amino acids region 1-312 tagged with Strep sequence at the C terminus	2
pMGKU416	pDONR201, *bscI* gene	This study
pMGKU417	pDONR201, *bscI* gene containing internal sequence-deletion and its flanking region	This study
pABB-CRS2	Suicide vector for conjugation, Amp^r	4
pMGKU418	pABB-CRS2, *bscI* gene containing internal sequence-deletion and its flanking region	This study
pABB-CRS2-*bspR*	pABB-CRS2, *bspR* gene containing internal sequence-deletion and its flanking region	5
pDONR-*bcr4*	pDONR201, *bcr4* gene for complementation	7
pMGKU419	pDONR201, *bcr4* gene tagged with FLAG sequence at the C terminus for complementation	This study
pMGKU420	pDONR201, *bcr4* gene lacking amino acids region 169-173 tagged with FLAG sequence at the C terminus	This study
pMGKU421	pDONR201, *bcr4* gene lacking amino acids region 164-173 tagged with FLAG sequence at the C terminus	This study
pMGKU422	pDONR201, *bcr4* gene lacking amino acids region 159-173 tagged with FLAG sequence at the C terminus	This study
pMGKU423	pDONR201, *bscI* gene for complementation	This study
pRK415-R4-R3-F	pRK415, recombination sites for MultiSite Gateway, Tet^r	8
pDONR-*fhaP*	pDONR-P4-P1R, *fha* promoter	8
pDONR-*rrnB*	pDONR-P2R-P3, *rrnB* terminator	8
pMGKU424	pRK415-R4-R3-F, *bcr4* gene tagged with FLAG sequence at the C terminus for complementation	This study
pMGKU425	pRK415-R4-R3-F, *bcr4* gene lacking amino acids region 169-173 tagged with FLAG sequence at the C terminus	This study
pMGKU426	pRK415-R4-R3-F, *bcr4* gene lacking amino acids region 164-173 tagged with FLAG sequence at the C terminus	This study
pMGKU427	pRK415-R4-R3-F, *bcr4* gene lacking amino acids region 159-173 tagged with FLAG sequence at the C terminus	This study
pMGKU428	pRK415-R4-R3-F, *bscI* gene	This study

Table S3. Primers used in the study	
Name	Sequence (5'→3')
B1-bscI-V5	AAAAAGCAGGCTTGTTGGACAGCGGACCCGCC
B2-bscI-V5	AGAAAGCTGGGTTTGACATTCTCGCCAGCGTGTC
B1-bscK-V5	AAAAAGCAGGCTTGGCGCACGCGCTGGTCCCG
B2-bscK-V5	AGAAAGCTGGGTTGGCTTGGGCGGGGAACGAGG
B1-BscJ-V5	AAAAAGCAGGCTTGGAGTACGAGTTGGTGGGC
B2-BscJ-V5	AGAAAGCTGGGTTGTCATGCCCGGCTCCTTCCG
B1-bcr4-comp	AAAAAGCAGGCTGCCAGGTCCGGTCTCGCACCG
B2-bcr4-V5	AGAAAGCTGGGTTTCCAGGAGCTCCAGGTAATG
5-HindIII-bcr4	ATCCGAATTCAAGCTTCATTCAGACTCAGGTTCAGATTC
3-bcr4-Strep	CGGGTGGCTCCATCCAGGAGCTCCAGGTAATG
5-HindIII-bscI	ATCCGAATTCAAGCTTAATTTGGATCTGACGGCGATC
3-bscI-Strep	CGGGTGGCTCCATGACATTCTCGCCAGCGTGTC
3-Strep-HindIII	GCAGGTCGACAAGCTTTCATTTTTCGAACTGCGGGTGGCTCCA
5-bcr4-58-109-Strep-IF	GCCGTCAGCGATCTGTCGGACTGGACGCCTGGTG
3-IF-bcr4-58-109-Strep	CAGATCGCTGACGGCCGCCG
5-bcr4-110-173-Strep-IF	TGGAGCCACCCGCAGTTCGA
3-IF-bcr4-110-173-Strep	CTGCGGGTGGCTCCAGCGGTCCTCCGCCTGTGCCG
B1-bscI	AAAAAGCAGGCTTCATGACTGTTCACGACGAC
B2-bscI	AGAAAGCTGGGTGCACAAGATCCAGCGCGACC
R1-bscI	CGACGTTGGCGTTGATCGCCGTCAGATC
R2-bscI	TCAACGCCAACGTCGACACGCTGGCGAG
5-bcr4-FLAG-IF	CCACCCGCAGTTCGAAAAATGAATTTGGATCTACCCAGCTTTCTTG
3-IF-bcr4-FLAG	TCGAACTGCGGGTGGCTCCATCCAGGACCTCCAGGTAATGGCTCG
3-IF-bcr4Δ169-173-FLAG	GTCATCCTTGTAGTCTAATGGCTCGGACTGCAACG
3-IF-bcr4Δ164-173-FLAG	GTCATCCTTGTAGTCCAACGGCGCGGGCCGCATCATG
3-IF-bcr4Δ159-173-FLAG	GTCATCCTTGTAGTCCATCATGGCGGGTCCGCTGTC
3-B2-bscI-comp	AGAAAGCTGGGTTTGACATTCTCGCCAGCGTGTC
5-bscI	CGATCTGCAGGCCAGGTT
3-bscI	CTATTGCCTTGCCCACCAA
5-bopD	CGGCTCGGTGAAGACATC
3-bopD	CCTCCCGCATCTGTTGAC
5-recA	ATGAAGATCGGCGTGATGT
3-recA	TAGAACTTGAGCGCGTTGC

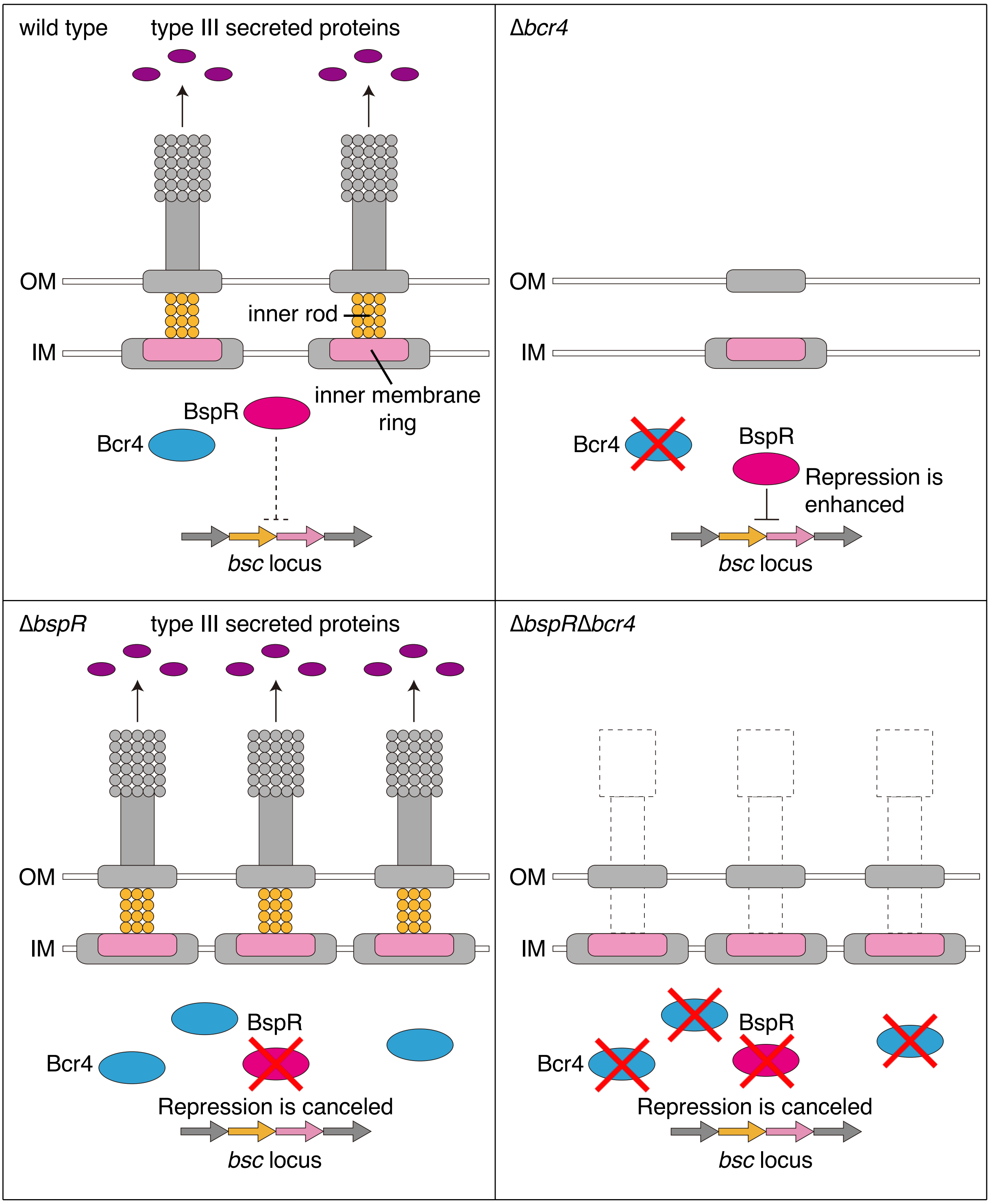

Fig. S1. Construction of the T3SS machinery in strains lacking Bcr4 and/or BspR. In the *B. bronchiseptica* wild-type (upper left), the BspR negative regulation level for the *bsc* locus transcription is moderate, and the T3SS machinery is established. In the Bcr4-deficient strain (upper right), BspR strongly represses the *bsc* locus transcription, and construction of the T3SS machinery is incomplete. In the BspR-deficient strain (lower left), the negative regulatory effect of BspR is cancelled, and the construction of the T3SS machinery is promoted. In the BspR/Bcr4 double-deficient strain (lower right), while the *bsc* locus transcription is promoted because of BspR deficiency, T3SS is not functional.

Fig. S1

```
Bb        1 MHSDSGSDSGSDSGSGS--PMASSIHPSEPIQPMEHVLEEADARLLTEVGFLAAAVSDLT     58
Bp        1 MHSDSGSDSGSDSGSGS--PMVSSIHPSEPIQPMEHVLEEADARLLTEVGFLAAAVSDLT     58
Bpp       1 MHSDSGSDSGSGSGSGSGSPMASSIHPSEPIQPMEHVLEEADARLLTEVGFLAAAVSDLT     60

Bb       59 RADAIFNALQRVRPGRTYPCIGLAVARMNAGLPDEAAEILANFQPAQAEDRSELDAWCGF    118
Bp       59 RADAIFNALQRVRPGRTHPCIGLAVARMNAGLPDEAAEILANFQPAQPEDRSELDAWCGF    118
Bpp      61 RADAIFNALQRVRPGRTYPCIGLAVARMNAGLPDEAAEVLANFQPAQAEDRSELDAWCGF    120

Bb      119 ALLLAGRSDEARRMLQRAIDAGGEAARLAQVVLDSGPAMMRPAPLQSEPLPGAPG         173
Bp      119 ALLLAGRSDEARRMLQRAIDAGGEAARLAQVVLDSGPAMMRPAPLQSEPLPGAPG         173
Bpp     121 ALLLAGRSDEARRMLQRAIDAGGEAARLAQVVLDSGPAMMRPAPLQSEPLPGAPG         175
```

Fig. S2. Alignment of Bcr4 amino acid sequences in representative *Bordetella* species. Bcr4 amino acid sequences of *B. bronchiseptica* S798 (Bb), *B. pertussis* Tohama I (Bp) and *B. parapertussis* 12822 (Bp) were compared using ClustalW. The grey highlighted letters represent amino acid residues that were different from those in Bb. Bcr4 of Bp and Bpp have 98.3% and 97.1% identities with those of Bp, respectively.

Fig. S2

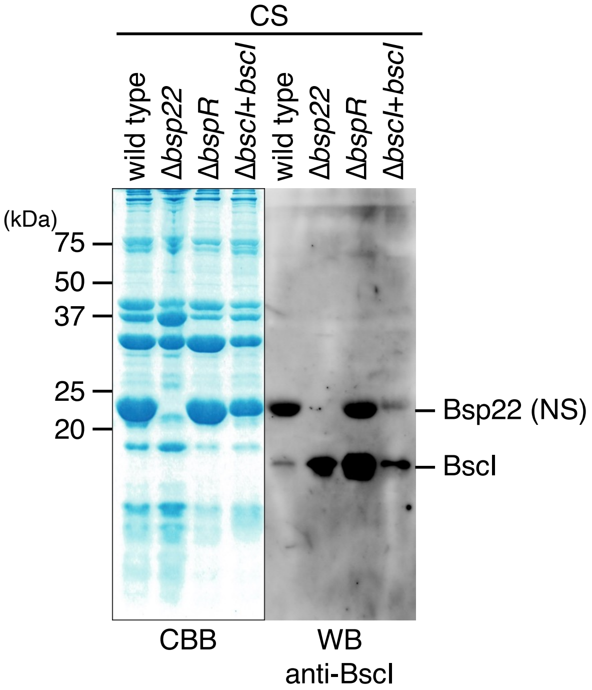

Fig. S3. The nonspecific reaction of anti-BscI antibody to Bsp22. The culture supernatants (CS) were prepared from the wild-type strain, Δ*bsp22* (Bsp22-deficient strain), Δ*bspR* (BspR-deficient strain) or Δ*bscI*+*bscI* (BscI-complemented strain) cultured in SS medium. The CS samples were separated by SDS-PAGE and stained with Coomassie Brilliant Blue (CBB, left panel) or analyzed by Western blotting (WB) with anti-BscI antibody (right panel). NS indicates nonspecific signals. Experiments were performed at least three times, and representative data are shown.

Fig. S3

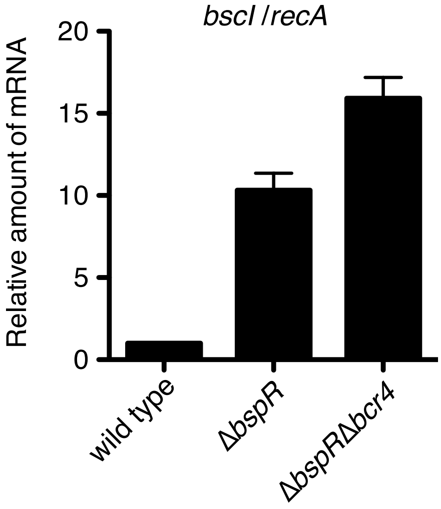

Fig. S4. The results of the RT-PCR analysis for the mRNA level of *bscI* in *B. bronchiseptica* strains. Total RNA was prepared from the wild-type strain, Δ*bspR* (BspR-deficient strain) or Δ*bspR*Δ*bcr4* (BspR- and Bcr4-deficient strain) cultured in SS medium and subjected to a quantitative RT-PCR analysis. The histogram shows the relative amount of *bscI* mRNA normalized by the housekeeping gene, *recA* mRNA. Experiments were performed at least three times, and representative data are shown.

Fig. S4

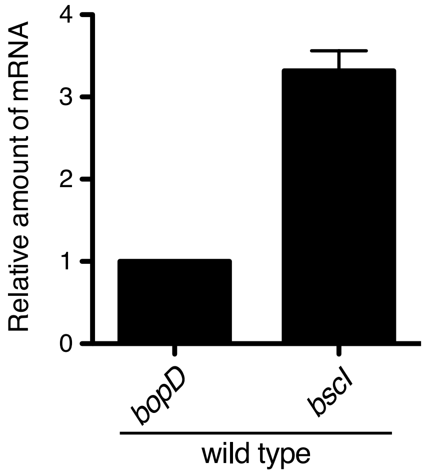

Fig. S5. The results of the RT-PCR analysis for mRNA levels of *bopD* and *bscI* in the wild-type *B. bronchiseptica*. Total RNA was prepared from the wild-type strain cultured in SS medium and subjected to a quantitative RT-PCR analysis. The histogram shows the relative amount of *bopD* and *bscI* mRNA in the wild-type. The relative ratio of *bscI* mRNA is shown when the *bopD* mRNA amount is set as 1. Experiments were performed at least three times, and representative data are shown.

Fig. S5

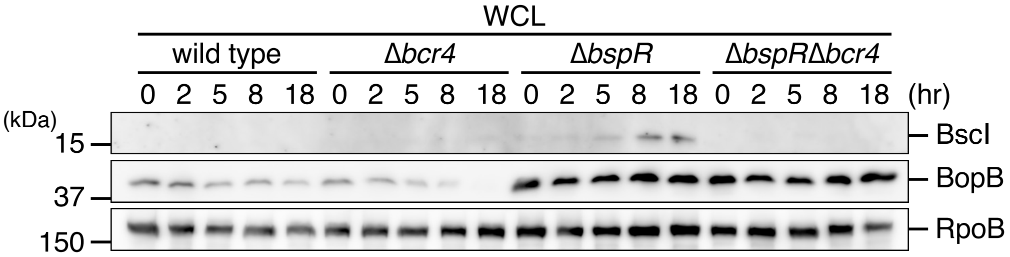

Fig. S6. The time course of BscI production in *B. bronchiseptica*. The whole cell lysates (WCL) were prepared from the wild-type strain, Δ*bcr4* (Bcr4-deficient strain), Δ*bspR* (BspR-deficient strain) or Δ*bspR*Δ*bcr4* (BspR- and Bcr4-deficient strain) cultured in SS medium for 0, 2, 5, 8 or 18 hr. The WCL were separated by SDS-PAGE and analyzed by Western blotting with antibodies against BscI, BopB and RpoB. Experiments were performed at least three times, and representative data are shown.

Fig. S6

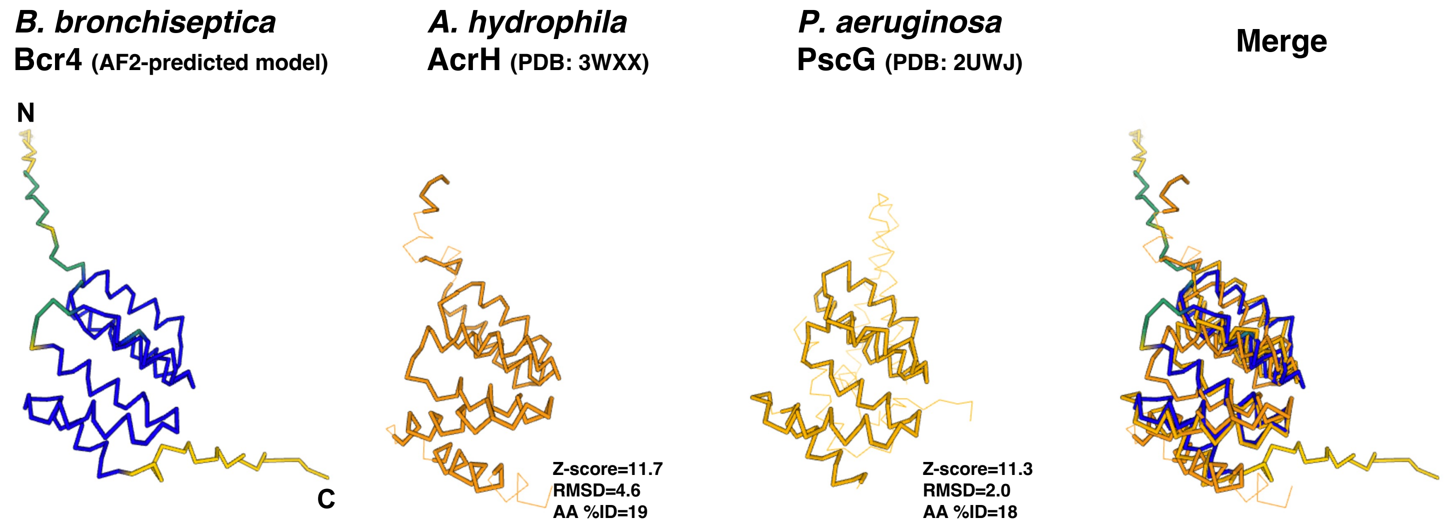

B

```
Bcr4 MHSDSGSDSGSDSGSGSPMASSIHPSEPIQPM-----EHVL-----EEADARLLTEVGFLAAAVSDLTRADAIFNALQRVRPGRTYPCIGLAVARMNA
AcrH -------------------------NEPAIEaflqdgGTLAxlndvSTDTLEQLYTLGFNQYHAGKHDEAHKIFQALCVLDHYEARFFLGLGACRQAL
PscG ----------------------------------------------DTSLIRELAELALAGSGQHCHEEALCIAEWLERLGQ-DEAARLIRISSLANQ

Bcr4 GLPDEAAEILANFQPAQAEDRSELDAWCGFALLLAGRSDEARRMLQRAIDAGG------EAARLAQVVLDSG-------PAMMRPAPLQSEPLPGAPG
AcrH GQFRLAIDSYSYGAXXDLQ-EPRFPFHAAECLLQLGELEGAESGFHSAQLLAAakpelaELAARAGIXLEVVktkkdxe-------------------
PscG GRYQEALAF-AHGNP-----WPALEPWFALCEWHLGLGAALDRRLAGLGGSSD-----pALADFAAGMRAQVr-------------------------

Bcr4 LLLLLLLLLLLLLLLLLLLLLLLLLLLLLLLL-----LLLL-----LHHHHHHHHHHHHHHHHLLLHHHHHHHHHHHHHHLLLLLHHHHHHHHHHHHL
AcrH -------------------------LLHHHHhhhhhlLLHHhhlllLHHHHHHHHHHHHHHHHLLLHHHHHHHHHHHHHHLLLLHHHHHHHHHHHHHL
PscG ----------------------------------------------LLHHHHHHHHHHHHHHLLLLHHHHHHHHHHHHHLLL-HHHHHHHHHHHHHHL

Bcr4 LLHHHHHHHHHHLLLLLHHHHHHHHHHHHHHHHHLLLHHHHHHHHHHHHHHLL------HHHHHHHHHHHHL-------HHHLLLLLLLLLLLLLLLL
AcrH LLHHHHHHHHHHHHHHLLL-LLHHHHHHHHHHHHHLLHHHHHHHHHHHHHHHHhlhhhhHHHHHHHHHHHHHhhhhhll-------------------
PscG LLHHHHHHH-HLLLL-----LHHHHHHHHHHHHHLLLHHHHHHHHHHHHLLLL-----hHHHHHHHHHHHHHl-------------------------
```

Fig. S7. The predicted structural model of *B. bronchiseptica* Bcr4

(A) The AlphaFold2 (AF2)-predicted structural model of *B. bronchiseptica* Bcr4 and structural comparison with *Aeromonas hydrophil*a AcrH (PDB: 3WXX) and *Pseudomonas aeruginosa* PscG (PDB: 2UWJ). Z-score, root mean square deviation (RMSD), and amino acid identity (AA %ID) of AcrH and PscG compared with Bcr4 are shown, respectively. (B) The pairwise sequence alignment of Bcr4, AcrH, and PscG. The most frequent amino acid type is colored. The secondary structure assignments (H/h: helix, E/e: strand, L/l: coil) are also shown.

Fig. S7
